# Typhoon Khanun-driven stormwater runoff enhances the detection of terrestrial mammalian environmental DNA in a forest stream

**DOI:** 10.3897/BDJ.14.e194007

**Published:** 2026-05-13

**Authors:** Keonhee Kim, Jung-Hwan Park

**Affiliations:** 1 Human and Ecocare Center, Konkuk University, Seoul, Republic of Korea Human and Ecocare Center, Konkuk University Seoul Republic of Korea; 2 PJ Factory. Co. Ltd, Seoul, Republic of Korea PJ Factory. Co. Ltd Seoul Republic of Korea

**Keywords:** environmental DNA, metabarcoding, storm runoff, urban stream, mammals

## Abstract

Monitoring terrestrial mammalian biodiversity remains challenging due to the limitations of conventional survey methods, including high labour requirements and detection bias. This study investigated how an extreme rainfall event, Typhoon Khanun (August 2023), influences the detection of terrestrial mammalian environmental DNA (eDNA) in a stream ecosystem. Samples were collected during the typhoon-induced runoff and again after hydrological conditions returned to baseline. Mammalian eDNA was successfully detected only during the rainfall event, whereas no mammalian DNA was detected in post-event samples. Metabarcoding analysis, targeting mitochondrial 12S rDNA, identified ten mammalian species from stormwater runoff, including both wild and anthropogenic taxa, while conventional surveys detected only two species at the same sites. These findings demonstrate that typhoon-driven runoff can transiently mobilise and concentrate terrestrial mammalian eDNA into stream systems, substantially enhancing detection probability. However, the detection of non-resident and anthropogenic species highlights the need for careful interpretation of runoff-derived eDNA signals. Overall, this study shows that extreme rainfall events can act as natural sampling windows that integrate catchment-scale biodiversity signals, providing a complementary approach for terrestrial mammal monitoring.

## Introduction

To date, the most commonly used methods for monitoring mammal distributions include physical evidence-based approaches, such as camera trapping, scat analysis and footprint tracking (Cromsigt et al. 2009, Fang et al. 2016, Khwaja et al. 2019, Kubo et al. 2019, Lee et al. 2019, Willcox et al. 2019). These techniques provide tangible evidence of mammal presence and valuable insights into their behaviour and habitat use. Camera traps can capture photos and videos of mammals active at specific times and locations, while faeces and footprints enable the indirect identification of species not captured on camera. However, in areas that are difficult to access or cover vast ranges, these methods can be highly inefficient (Prosekov et al. 2020). Additionally, camera installation, scat collection and track surveys demand substantial time and resources. Environmental conditions can rapidly erase traces, complicating accurate status assessments and species identification (Cutler and Swann 1999, Newey et al. 2015, Richardson et al. 2018).

Environmental DNA (eDNA) approaches to biodiversity monitoring have emerged as effective alternatives to traditional physical methods, overcoming some of their limitations and providing high efficiency for mammal surveys (Beng and Corlett 2020, Harper et al. 2019, Rose et al. 2019). eDNA consists of genetic material shed by organisms into the environment, enabling the indirect detection of target taxa through genetic analysis (Othman et al. 2021). As eDNA can be extracted from various environmental samples, such as water and soil, allowing for the selective identification of target species, this makes it a fast and accurate tool for assessing species presence. (Bohmann et al. 2014, Othman et al. 2023). In particular, eDNA has been widely applied to aquatic ecosystems, such as rivers and wetlands, to non-invasively identify fish and macroinvertebrate diversity (Senapati et al. 2019, Lyet et al. 2021). Given its applicability to a wide range of taxa and environments, the utility of eDNA continues to grow. In mammalian studies, eDNA has primarily been used to analyse water collected from natural streams or artificially constructed ponds to detect mammalian DNA (Sales et al. 2020, Girolamo 2022, Broadhurs et al. 2025). However, these studies have often failed to capture the full species richness of the target areas. One reason for this is the challenge of concentrating eDNA from the terrestrial environment into limited waterbodies, such as streams or ponds, which leads to low detection efficiency (Ushio et al. 2017, Harper et al. 2019, Chacko et al. 2023).

Despite the growing application of eDNA for terrestrial mammal detection, the influence of extreme hydrological events on eDNA transport and detectability remains poorly understood (Tangil and Rodriguez 2021). In particular, heavy rainfall events, such as typhoons, can generate intense surface runoff, potentially mobilising terrestrial-derived DNA from across the catchment into stream systems (Khaleel et al. 1980, Waterloo et al. 2006, Göbel et al. 2007, Danacova et al. 2017). This process may temporarily enhance the detectability of mammalian biodiversity beyond what is achievable under baseline conditions, yet it has rarely been explicitly examined in terrestrial eDNA studies.

In this study, we take advantage of Typhoon Khanun as a natural hydrological disturbance event to evaluate how rainfall-driven runoff influences the detection of terrestrial mammalian eDNA in a mountainous forest stream. Specifically, we: (1) compare mammalian eDNA detection during and after the typhoon event; (2) assess the role of runoff as a transport mechanism for terrestrial DNA into aquatic systems and (3) evaluate the consistency between eDNA-based detection and conventional long-term monitoring records. By explicitly incorporating an extreme rainfall event into the study design, this work aims to clarify the ecological and methodological implications of event-driven eDNA signals in terrestrial biodiversity monitoring.

## Materials and Methods

### Study region

**Figure 1.**
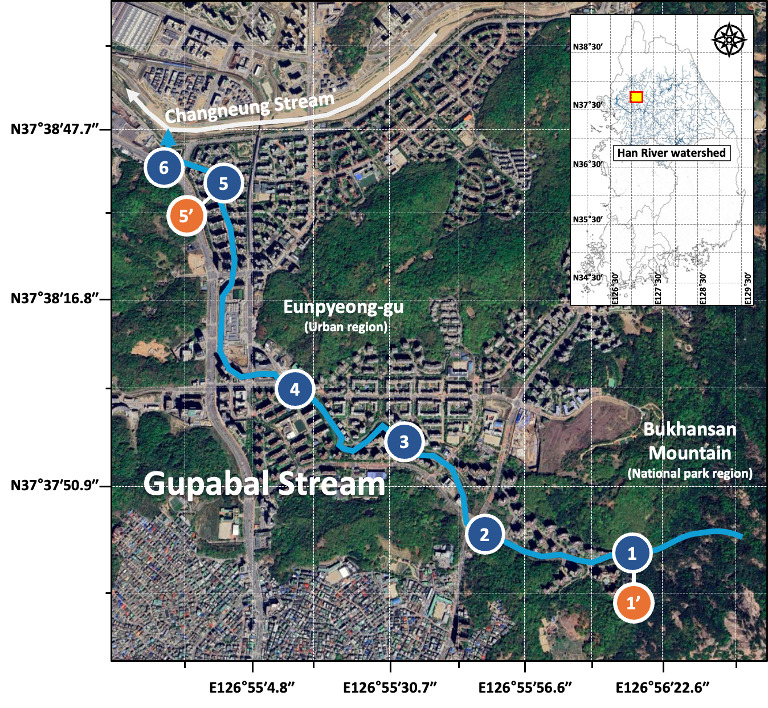
Study area and sampling locations along the Gupabal Stream. Sampling was conducted during Typhoon Khanun (10-11 August 2023) at stormwater inflow sites (Sites 1′ and 5′) and after the runoff had subsided (26 August 2023) at six sites along the stream under baseline conditions.

This study was conducted along a 3.43 km section of the Gupabal Stream, which originates in a mountainous forest area bordering northern Seoul (Fig. 1). The stream begins near the Seonlimsa Temple on the western slope of Bukhansan Mountain, at an elevation of approximately 100 metres in Jingwan-dong, Eunpyeong, Seoul. It flows through the New Town of Eunpyeong before merging with the Changneung Stream. The surrounding area features various recreational facilities, including walking paths and exercise equipment and artificial water inflow is utilised to maintain a constant stream depth of approximately 0.2 m. (Lee 2022). During the study period in August 2023, Seoul received a cumulative monthly precipitation of 298.1 mm, with the highest rainfall occurring on the 10th and 11th, totalling 86.1 mm.

The headwaters of the stream are located within a nationally protected area that stretches between Seoul and the neighbouring Gyeonggi Province, which includes a major urban park that spans northern Seoul and extends into Goyang and Uijeongbu in Gyeonggi Province. This park is home to more than 1,300 species of flora and fauna, but it is also subject to intense human activity, including noise, stream pollution, illegal harvesting of forest products and the proliferation of unauthorised trails. These factors contribute to habitat disturbance and degradation for wildlife (Doh et al. 1986, Kim et al. 1987, Kang and Sung 2016).

### eDNA sampling and extraction

Environmental DNA (eDNA) sampling was initially planned at six sites where runoff from Bukhansan National Park enters the Gupabal Stream. However, due to Typhoon Khanun (10-11 August 2023; cumulative rainfall of approximately 86.1 mm in Seoul), storm-flow samples could only be collected at Sites 1 (upstream) and 5 (downstream). Field-replicated storm-runoff samples were collected at these sites when rainfall intensity exceeded approximately 10–20 mm/h. After the typhoon had passed and stormwater runoff had completely receded by 26 August, water samples were collected along the Gupabal Stream once turbidity levels returned to baseline (pre-rain) conditions.

This sampling design explicitly contrasts event-driven hydrological conditions during the typhoon with post-event baseline conditions.

Sampling was conducted twice at each site using plastic beakers suspended from a bridge by ropes. Collected water was transferred to 2 litre sampling bottles and transported to the laboratory within 12 hours.

In the laboratory, water samples were filtered through GF/F glass fibre filters (Whatman) until clogging occurred, with a minimum of 500 ml filtered per sample. Filters were then placed in Salivette tubes (SARSTEDT, Nümbrecht, Germany) and stored at −80°C until DNA extraction.

eDNA was extracted following the protocol outlined by Minamoto et al. (2021), using the DNeasy Blood & Tissue Kit (Qiagen, Hilden, Germany). The extraction process involved cell lysis on the GF/F filters using Buffer ATL, Proteinase K and Buffer AL, followed by DNA purification. A final elution volume of 100 µl of purified eDNA was obtained.

### PCR amplification and Metabarcoding

Mammalian DNA in stormwater runoff samples was amplified targeting the mitochondrial 12S rDNA region using the MiMammal-U primer set (Ushio et al. 2017). PCR reactions were performed with 2× GainBlue Hot Start Max PCR Master Mix™ (Gainbio, Korea), with primers at 10 pmol/µl and 3 µl of template eDNA per reaction. Thermal cycling conditions consisted of an initial denaturation at 95°C for 3 min, followed by 35 cycles of 95°C for 10 s, 65°C for 20 s and 72°C for 20 s, with a final extension at 72°C for 5 min. Amplicons were visualised on 2% agarose gels and target bands were excised and purified. Amplicons were visualised on 2% agarose E-Gel system 'SizeSelect gels' (Thermo Fisher Scientific, Waltham, MA, USA) and target fragments of the expected size were recovered according to the manufacturer’s protocol. PCR reactions were performed in duplicate for each sample and replicate amplicons were pooled prior to library preparation to minimise amplification bias.

To minimise amplification bias, first-round PCR was conducted in duplicate and pooled prior to library preparation. A negative control (nuclease-free water) was included and processed identically to monitor contamination.

Purified amplicons were used for library preparation and sequenced using paired-end Illumina sequencing at Macrogen Inc. (Seoul, Republic of Korea), following the manufacturer’s standard protocols. Demultiplexed reads were processed with Cutadapt (Martin 2011) to remove primer sequences and amplicon sequence variants (ASVs) were inferred using DADA2 v.1.22 (Callahan et al. 2016), including quality filtering, chimera removal and exclusion of non-target sequences.

Taxonomic assignment was performed using a curated mitochondrial 12S reference database (NCBI nt filtered to Mammalia) with a BLASTn-based lowest common ancestor (LCA) approach. Species-level identification was assigned at ≥ 98% sequence identity and ≥ 100 bp alignment length; otherwise, reads were assigned to higher taxonomic levels. Non-mammalian reads and potential contaminants were removed, based on negative controls and read thresholds prior to downstream analyses. For each site, reads from two field replicates were pooled and used to calculate species-level relative read abundance.

### Statistical analysis

All statistical analyses were performed in R (version 4.5.1) (Chambers 2008). Mammalian read counts were log10-transformed before analysis to minimise the influence of highly abundant taxa. Dissimilarities in community composition between samples were calculated using the Bray–Curtis index. Hierarchical clustering was conducted using the complete linkage method, based on these dissimilarities. Unless otherwise noted, species-level read proportions were calculated using only non-human mammalian reads.

### Verifying eDNA detection

To verify the mammalian species detected in the eDNA samples from Gupabal Stream, we compared the results with the long-term mammal monitoring data collected regularly in Bukhansan National Park (Natural Resource Survey of Bukhansan National Park) (Kim et al. 2021). We also reviewed previously published literature on mammal surveys conducted in Bukhansan National Park to identify the known diversity of mammalian species in the area and compared these records with our eDNA results (Kim et al. 2023, Korea National Park Service 2023).

## Data resources

The data that support the findings of this study are available in NCBI Sequence Read Archive (SRA) at https://www.ncbi.nlm.nih.gov/, reference number PRJNA1427895. These data were derived from the following resources available in the public domain: NCBI SRA, https://dataview.ncbi.nlm.nih.gov/object/PRJNA1427895?reviewer=rbg6sbrlht3peocg10bjugt59f.

## Results

### Diversity of mitochondrial 12S rDNA gene

Mammalian eDNA detection was strongly dependent on hydrological conditions associated with the typhoon event. Mitochondrial 12S rDNA was successfully amplified from all stormwater runoff samples collected during Typhoon Khanun at both upstream (Site 1) and downstream (Site 5) locations (Fig. 2). In contrast, no mammalian eDNA was detected in any samples collected after runoff had subsided, despite sampling across six sites along the stream. This clear contrast indicates that mammalian eDNA detection in this system was transient and primarily driven by rainfall-induced runoff rather than persistent background DNA in the stream.

Metabarcoding analysis of the stormwater runoff samples collected during the rainfall event identified a total of 58 amplicon sequence variants (ASVs) from 198,929 reads, all assigned to the Chordata phylum (Fig. 3). At Site 1 (near the upper watershed adjacent to Bukhansan National Park), 91,042 reads corresponding to 33 ASVs were detected, while at Site 5 (located within a residential apartment complex), 107,887 reads representing 39 ASVs were identified. Amongst these, 14 ASVs were shared between the two sites, totalling 185,498 reads. Most ASVs yielded more than 10 reads, with an average of approximately 3,430 reads per ASV. However, the number of reads per ASV varied widely, with a standard deviation of ± 9,741 reads, indicating high variability in amplification between ASVs.

### Mammalian DNA diver

Taxonomic analysis of mitochondrial 12S rDNA sequences from Sites 1 and 5 revealed that primates were responsible for 86% and 82%, respectively, assigned to *Homo sapiens*. After excluding *Homo sapiens*, mammalian sequences detected at Sites 1 and 2 accounted for 14% and 18% of the total sequences, respectively and consisted of taxa from the orders Artiodactyla, Rodentia, Eulipotyphla and Carnivora. Following removal of human ASV, 12 mammalian species were detected. Amongst them, *Bos taurus* (Domestic Cattle) was the most abundant species, accounting for 25.3%, followed by *Mus musculus* (House Mouse) (17.5%) and *Sus scrofa* (Eurasian Wild Pig) (17.1%) (Fig. 4) (Suppl. material 1). *Mogera robusta* (Large Mole), *Canis lupus familiaris* (Domestic Dog), *Sciurus vulgaris* (Red Squirrel) and *Rattus norvegicus* (Brown Rat) were other frequently detected species (> 5%), whereas *Felis catus* (Domestic Cat), *Meles leucurus* (Asian Badger), *Apodemus agrarius* (Striped Field Mouse), *Hydropotes inermis* (Water Deer) and *Ovis aries* (Sheep) were detected at much lower abundances (< 2%), with *H. inermis*, *A. agrarius* and *O. aries* each contributing < 1%. *M. musculus* was the most abundant species.

When comparing the results upstream (Site 1) and downstream (Site 5) of mammalian species, 10 mammalian species were identified from 12,238 reads at Site 1 (32.8%), followed by *S. scrofa* (30.7%) and *S. vulgaris* (11.0%). Other detected species included *C. lupus familiaris* (6.3%), *R. norvegicus* (4.7%), *B. taurus* (3.9%), *M. leucurus* (3.3%), *F. catus* (2.9%), *M. robusta* (2.3%) and *A. agrarius* (2.2%). In contrast, 11 mammalian species were detected at Site 5 from 18,854 reads — reater than the total read count from Site 1. At this site, *B. taurus* dominated the community with 39.2% of non-human mammalian reads. *M. robusta* and *C. lupus familiaris* were also more abundant, accounting for 19.6% and 14.1% of reads, respectively — representing 8- to 10-fold increases compared to Site 1. Additionally, *H. inermis* (0.2%) and *O. aries* (0.4%) were detected only at Site 5 and not upstream.

**Figure 5.**
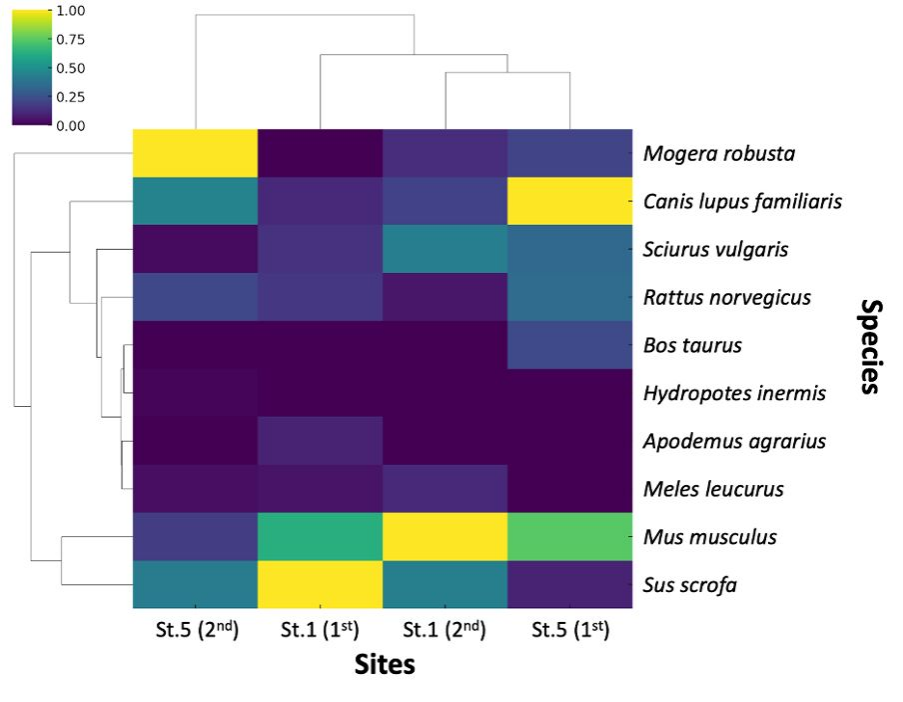
Hierarchical clustering heatmap of non-human mammalian eDNA reads from stormwater runoff samples collected during Typhoon Khanun (10-11 August 2023). Columns represent field replicates from Site 1 (St.1–Rep1, Rep2) and Site 5 (St.5–Rep1, Rep2), and rows represent detected mammalian species.

Consistent with the observed differences in species composition and abundance, the hierarchical clustering heatmap (Fig. 5) illustrated clear differences in non-human mammalian eDNA composition across sites. Replicates from Site 1 (St.1–Rep1, St.1–Rep2) clustered together and were distinct from those from Site 5 (St.5–Rep1, St.5–Rep2), indicating consistent similarity within sites and differentiation between them (Suppl. material 1). At the species level, domestic taxa, such as *Bos taurus*, formed a separate cluster from wild small mammals like *Mus musculus*, *Rattus norvegicus* and *Apodemus agrarius*. Forest-associated species, including *Mogera robusta*, *Sciurus vulgaris* and *Meles leucurus*, grouped together, while *Sus scrofa* appeared as an independent branch, suggesting a unique distribution pattern. These findings demonstrate that stormwater runoff eDNA captured both livestock-associated and native forest mammals, reflecting distinct community structures upstream and downstream.

### Verifying mammalian diversity in the upper waters

To evaluate the reliability of mammalian species identified by environmental DNA (eDNA), the findings were compared with long-term monitoring data from the Bukhansan National Park Natural Resource Survey (Fig. 6). Traditional monitoring at 26 sites across the Park documented nine mammalian species through direct observation and camera trapping (Fig. 6a). These species included *H. inermis*, *S. scrofa*, *C. lupus familiaris*, *Nyctereutes procyonoides*, *F. catus*, *M. leucurus*, *Mustela nivalis*, *Eutamias sibiricus* and *S. vulgaris*. Of these, only *H. inermis* and *S. scrofa* were detected near the eDNA sampling sites.

From the nine species identified through conventional monitoring, six were also detected by eDNA: *H. inermis*, *S. scrofa*, *C. lupus familiaris*, *F. catus*, *M. leucurus* and *S. vulgaris* (Fig. 6b). However, several rodent species identified through eDNA — such as *M. musculus*, *M. robusta*, *R. norvegicus* and *A. agrarius* — were not observed in the traditional survey, either as individuals or through trace evidence.

Additionally, DNA from *B. taurus* and *O. aries* was found in the eDNA samples, despite the absence of records indicating that cattle or sheep are raised or pastured within Bukhansan National Park. Currently, there are no facilities or reports of livestock farming involving these species in the area. However, the Park experiences high levels of human visitation and nearby downstream areas feature numerous restaurants selling lamb and beef close to the stream.

## Discussion

The complete absence of mammalian eDNA in post-event samples, despite detection during the typhoon, indicates that eDNA signals in this system were strongly dependent on rainfall-driven transport processes. This finding suggests that the detected DNA did not originate from stable, local sources within the stream, but rather from transient inputs mobilised from the surrounding terrestrial environment. During intense rainfall, surface runoff can entrain biological materials such as faeces, hair and epithelial cells from across the catchment and rapidly transport them into stream networks. Consequently, extreme hydrological events, such as typhoons, can act as dominant drivers of eDNA availability in lotic systems.

Building on this transport-driven mechanism, storm-driven runoff events may function as temporary integrators of biodiversity signals across the landscape. Unlike conventional sampling under baseflow conditions, which reflects localised and often limited DNA sources, event-based runoff sampling captures a broader spatial footprint of terrestrial activity within the catchment. This mechanism may explain the higher diversity of mammalian taxa detected in this study compared to conventional monitoring methods. Therefore, extreme rainfall events can provide unique opportunities to obtain integrated, catchment-scale biodiversity information that would otherwise remain undetected.

However, this transport-driven integration also introduces important limitations. As the detected DNA is transported from multiple upstream sources, it does not necessarily indicate the local presence or abundance of organisms at the sampling site. In addition, metabarcoding read counts are influenced by multiple factors, including primer bias, PCR stochasticity, DNA degradation and sequencing depth and, therefore, cannot be directly interpreted as quantitative estimates of species abundance. In this study, log-transformed read counts were used to reduce the influence of highly dominant taxa and to facilitate comparison amongst samples. Accordingly, read counts were interpreted only as relative eDNA signals amongst samples and not as direct estimates of species abundance and runoff-derived eDNA should be considered a qualitative or semi-quantitative indicator of biodiversity rather than a direct measure of population size or density.

Despite these limitations, stormwater runoff-derived eDNA still demonstrated considerable potential for assessing mammalian diversity in forested ecosystems. In particular, the approach enabled the detection of a broader range of taxa than conventional monitoring methods, including small-bodied mammals, such as *Mus musculus*, *Rattus norvegicus* and *Mogera robusta*, which are typically difficult to detect using camera traps or faecal surveys. These findings highlight that runoff-derived eDNA can serve as an effective complementary tool for capturing otherwise overlooked components of mammalian biodiversity.

A large proportion of sequence reads were assigned to *Homo sapiens*, likely reflecting the strong influence of human activity within the study area. Although human-derived DNA is commonly detected in environmental samples, its interpretation requires caution because such signals may originate from various anthropogenic sources. Both upstream and downstream sites detected eDNA from *B. taurus* (Artiodactyla), with relative abundance values more than twice as high at the downstream site. Notably, *B. taurus* DNA was over ten times more abundant downstream compared to upstream. Since livestock grazing does not occur in the national park, the detected *B. taurus* DNA likely does not indicate the presence of resident animals. Previous research has shown that even thermally processed meat products can retain trace amounts of amplifiable DNA (Arslan et al. 2006, Murray et al. 2007, Şakalar et al. 2012, Pollack et al. 2018), suggesting that incidental human activities, such as food consumption by visitors, may contribute to these signals. Thus, the detection of *B. taurus* DNA suggests that stormwater runoff-derived eDNA can capture non-target genetic signals associated with transient human use of forested landscapes, warranting cautious interpretation in biodiversity assessments.

DNA from Ovis aries was detected at downstream sites despite the absence of livestock farming in Bukhansan National Park and its surrounding areas (Kim et al. 2021, Korea National Park Service 2023). This signal is, therefore, unlikely to reflect local presence and more likely originates from anthropogenic inputs introduced into the system and transported via stormwater runoff (Shogren et al. 2017, Calderón-Franco et al. 2021). Although taxonomic misassignment due to database limitations cannot be fully excluded, it is considered less likely in this ecological context.

Three mammal species recorded in conventional long-term monitoring programmes — *N. procyonoides*, *M. sibirica* and *E. sibiricus* — were not found in the runoff-derived eDNA samples. This discrepancy likely reflects methodological differences between the two monitoring approaches rather than simple detection failure; rather, it highlights fundamental differences in how the two approaches capture species presence. Conventional monitoring assesses species presence over extended periods, while stormwater runoff eDNA primarily reflects genetic material collected during rainfall events. As a result, species with relatively stable, but low recent activity near runoff pathways, may be under-represented in event-based eDNA samples. For instance, although *H. inermis* demonstrated relatively high densities in conventional surveys (Kim et al. 2021), it was not detected at the upstream eDNA site and was found only at low levels downstream. This pattern suggests that the success of detecting runoff eDNA is influenced not only by population size, but also by the timing of species activity in relation to rainfall and hydrological transport processes. Therefore, optimising sampling timing and increasing replication across rainfall events may enhance detection sensitivity for certain species. Future studies incorporating repeated sampling across multiple rainfall events will help clarify the consistency of runoff-derived eDNA signals and further refine event-based biodiversity monitoring strategies in forested catchments.

## Conclusions

Stormwater runoff eDNA is an effective method for assessing mammal diversity in mountain forest ecosystems, especially in areas that are difficult to access for field surveys. While eDNA-based biodiversity monitoring offers significant advantages, such as being non-invasive and highly efficient, it has limitations. It lacks direct observation of organisms and struggles to differentiate incidental external DNA inputs. Due to this challenge — specifically, the difficulty in distinguishing allochthonous DNA — eDNA cannot fully replace traditional monitoring methods that rely on direct observation. Instead, it should be viewed as a complementary tool. Despite these constraints, the high efficiency of eDNA for monitoring biodiversity over large spatial areas could lead to a shift in monitoring strategies from observation-centred surveys to eDNA-based approaches. In this context, the results from eDNA monitoring can provide an evidence-based foundation to guide subsequent field surveys, acting as a signal to determine when and where direct observation-based monitoring should occur.

## Supplementary Material

3A823990-0804-5CEB-9623-DC022CD90E78 10.3897/BDJ.14.e194007.suppl1 Supplementary material 1 Filtered mammalian sequence data Data type metabarcoding read number Brief description Filtered mammalian sequence data derived from the raw reads deposited in the NCBI Sequence Read Archive (SRA; BioProject accession number PRJNA1427895). Only reads assigned to Mammalia, based on BLASTn analysis (≥ 98% sequence identity and ≥ 100 bp alignment length) are included. The table provides species-level taxonomic assignments and corresponding read counts for each site and their replicates. File: oo_1573277.docx https://binary.pensoft.net/file/1573277 Keonhee Kim

## Figures and Tables

**Figure 2a.**
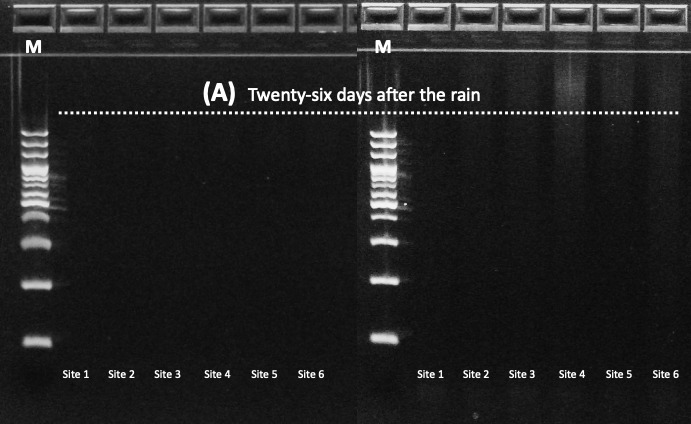
Samples collected under baseline conditions 26 days after Typhoon Khanun (26 August 2023) showing no amplification across Sites 1–6;

**Figure 2b.**
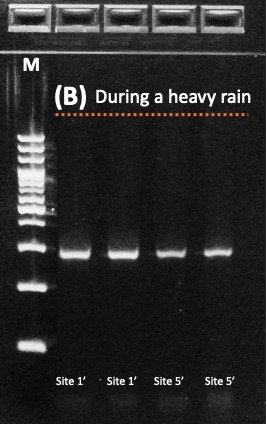
Samples collected during typhoon-induced runoff (10-11 August 2023) at Sites 1′ and 5′ showing successful amplification of mammalian eDNA.

**Figure 3a.**
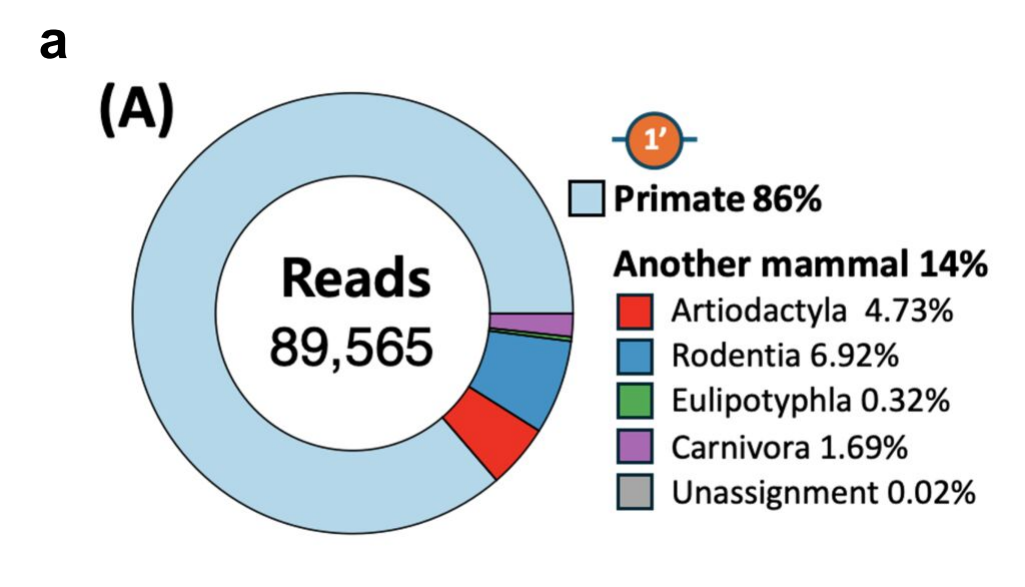
(a) Site 1 (upstream);

**Figure 3b.**
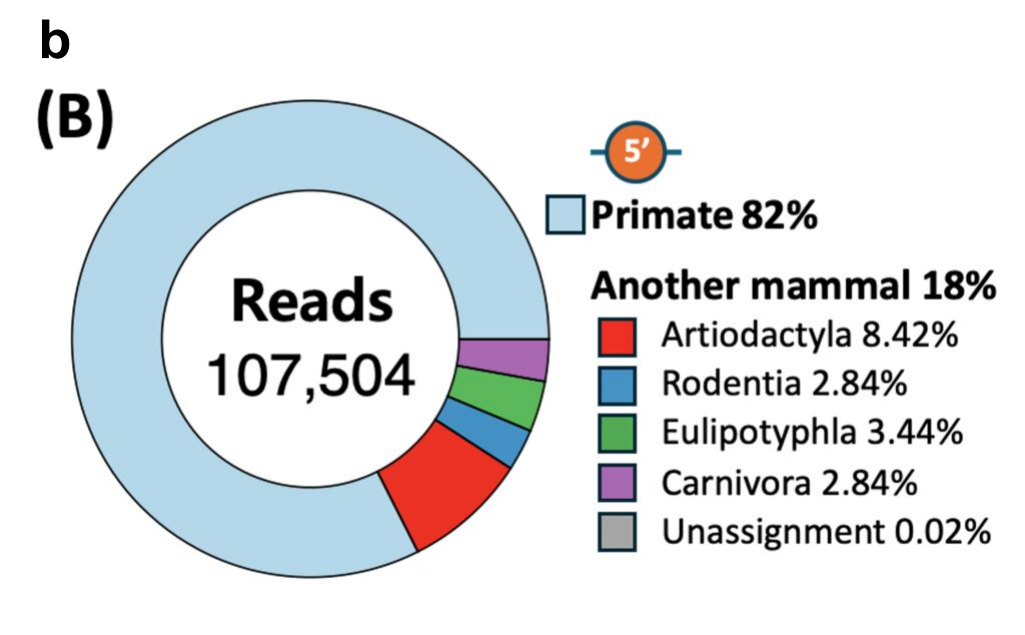
(b) Site 5 (downstream).

**Figure 4a.**
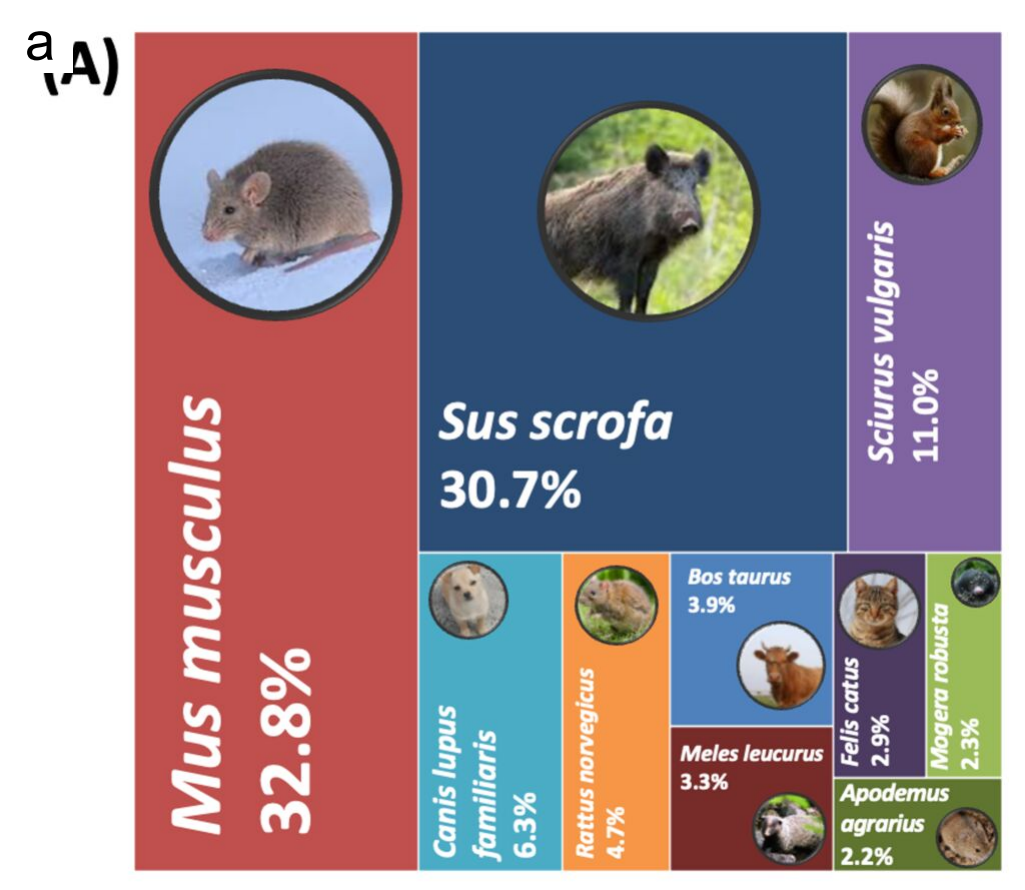
Site 1 (upstream);

**Figure 4b.**
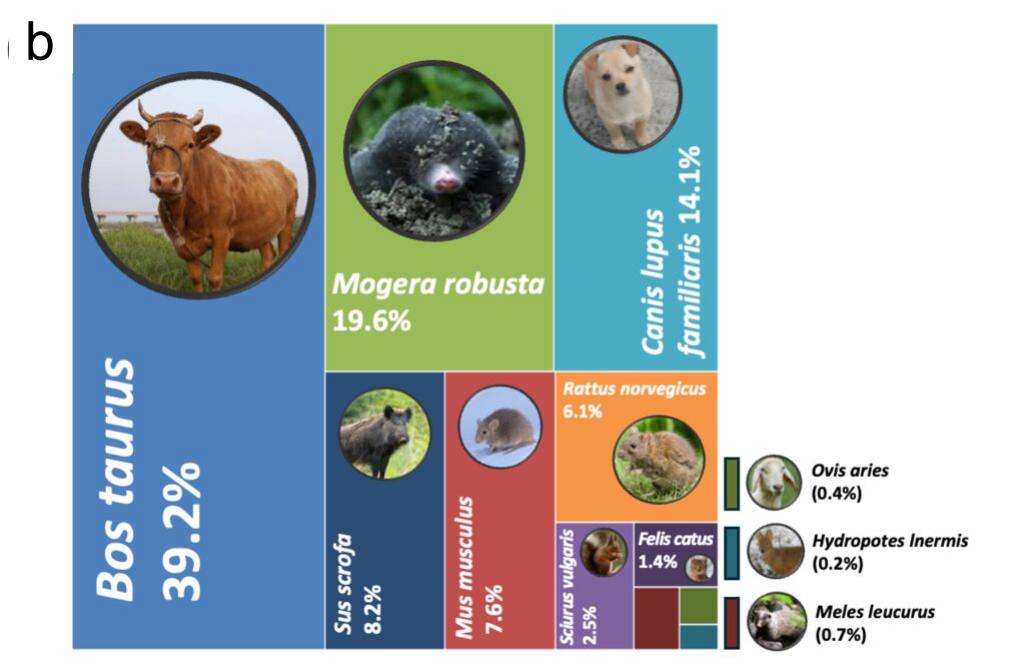
Site 5 (downstream).

**Figure 6a.**
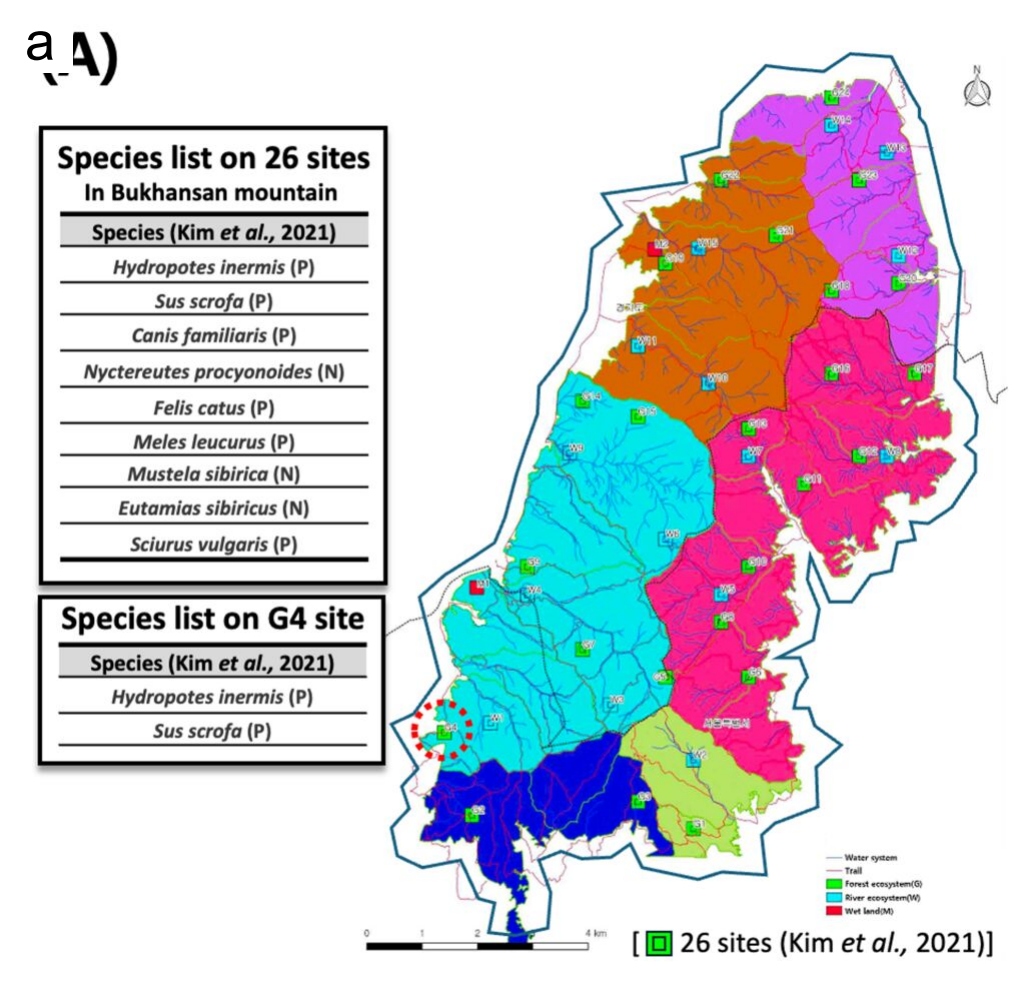
Spatial distribution of camera-trap survey sites and recorded species (Kim et al. 2021). Coloured squares indicate camera-trap locations and associated habitat types (red: wetland; green: forest ecosystem; light blue: river ecosystem), adapted from Kim et al. (2021). The study area is divided into five survey regions;

**Figure 6b.**
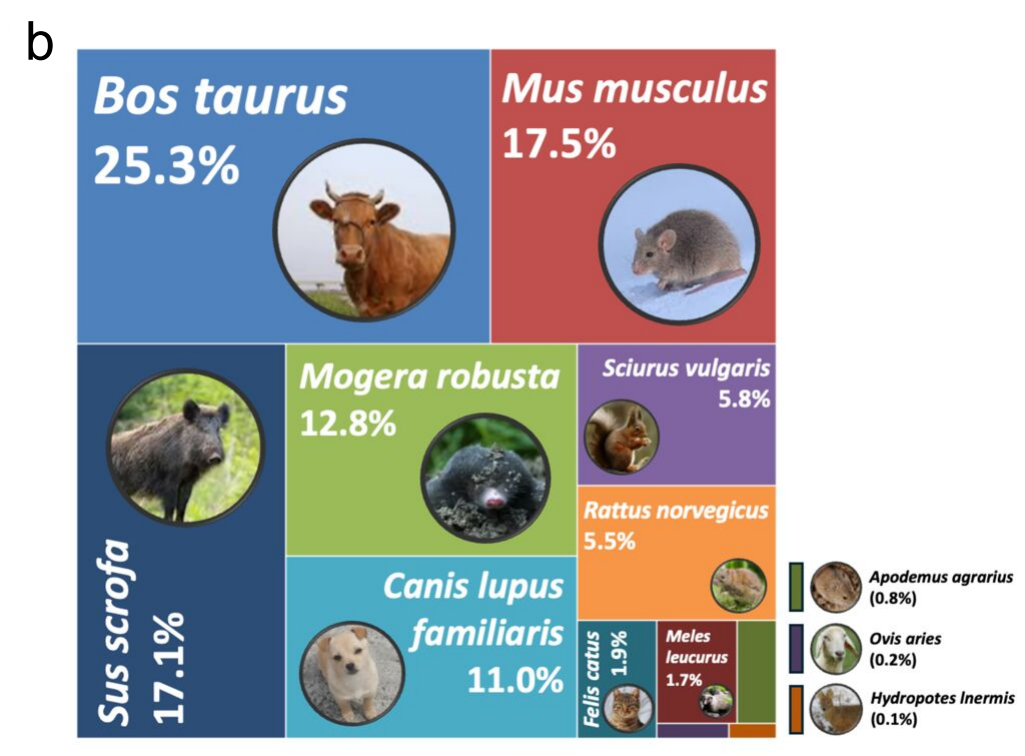
Relative read abundance of mammalian species detected by eDNA metabarcoding.
