# Supplementary material for: Typhoon Khanun-driven stormwater runoff enhances the detection of terrestrial mammalian environmental DNA in a forest stream
Source: Biodivers Data J. 2026 May 13;14:e194007. doi: 10.3897/BDJ.14.e194007 (PMC13191331; doi:10.3897/BDJ.14.e194007)
Supplement: Supplementary material 1 — Filtered mammalian sequence data [file bdj-14-e194007-s001.docx]

**Table S1.** Filtered mammalian sequence data derived from the raw reads deposited in the NCBI Sequence Read Archive (SRA; BioProject accession number PRJNA1427895). Only reads assigned to Mammalia based on BLASTn analysis (≥98% sequence identity and ≥100 bp alignment length) are included. The table provides species-level taxonomic assignments and corresponding read counts for each site and replicate.

| M1-12S | M2-12S | M3-12S | M4-12S | Class | Order | Family | Genus | Species | |
| --- | --- | --- | --- | --- | --- | --- | --- | --- | --- |
| 38,374 | 36,285 | 42,221 | 31,352 | Mammalia | Primates | Hominidae | *Homo* | | *Human DNA* |
| 1130 | 0 | 992 | 5,450 | Mammalia | Primates | Hominidae | *Homo* | | *Human DNA* |
| 476 | 0 | 4,238 | 2,735 | Mammalia | Artiodactyla | Bovidae | *Bos* | | *-* |
| 0 | 400 | 5,202 | 400 | Mammalia | Primates | Hominidae | *Homo* | | *Human DNA* |
| 1,657 | 2,238 | 794 | 613 | Mammalia | Rodentia | Muridae | *Mus* | | *Mus musculus* |
| 0 | 287 | 224 | 3,473 | Mammalia | Eulipotyphla | Talpidae | *Mogera* | | *Mogera robusta* |
| 0 | 0 | 1,900 | 382 | Mammalia | Primates | Hominidae | *Homo* | | *Homo sapiens* |
| 334 | 950 | 371 | 97 | Mammalia | Rodentia | Sciuridae | *Sciurus* | | *Sciurus vulgaris* |
| 0 | 0 | 612 | 1,136 | Mammalia | Carnivora | Canidae | *Canis* | | *Canis lupus* |
| 442 | 138 | 388 | 755 | Mammalia | Rodentia | Muridae | *Rattus* | | *Rattus norvegicus* |
| 1,572 | 135 | 0 | 0 | Mammalia | Artiodactyla | Suidae | *Sus* | | *Sus scrofa* |
| 0 | 0 | 0 | 1,447 | Mammalia | Artiodactyla | Suidae | *Sus* | | *Sus scrofa* |
| 324 | 387 | 309 | 421 | Mammalia | Carnivora | Canidae | *Canis* | | *Canis lupus* |
| 1,110 | 0 | 0 | 0 | Mammalia | Artiodactyla | Suidae | *Sus* | | *Sus scrofa* |
| 0 | 819 | 105 | 0 | Mammalia | Artiodactyla | Suidae | *Sus* | | *Sus scrofa* |
| 143 | 256 | 0 | 133 | Mammalia | Carnivora | Mustelidae | *Meles* | | *Meles leucurus* |
| 91 | 259 | 0 | 0 | Mammalia | Carnivora | Felidae | *Felis* | | *-* |
| 0 | 350 | 0 | 0 | Mammalia | Primates | Hominidae | *Homo* | | *Homo sapiens* |
| 0 | 0 | 0 | 344 | Mammalia | Primates | Hominidae | *Homo* | | *Homo sapiens* |
| 0 | 325 | 0 | 0 | Mammalia | Primates | Hominidae | *Homo* | | *Homo sapiens* |
| 264 | 0 | 0 | 0 | Mammalia | Rodentia | Muridae | *Apodemus* | | *Apodemus agrarius* |
| 0 | 141 | 113 | 0 | Mammalia | Primates | Hominidae | *Homo* | | *Human DNA* |
| 0 | 0 | 0 | 193 | Mammalia | Carnivora | Felidae | *Felis* | | *-* |
| 0 | 0 | 189 | 0 | Mammalia | Carnivora | Canidae | *Canis* | | *Canis lupus* |
| 0 | 0 | 166 | 0 | Mammalia | Artiodactyla | Bovidae | *Bos* | | *-* |
| 0 | 0 | 148 | 0 | Mammalia | Primates | Hominidae | *Homo* | | *Human DNA* |
| 135 | 0 | 0 | 0 | Mammalia | Primates | - | *Homo* | | *Human DNA* |
| 0 | 0 | 134 | 0 | Mammalia | Artiodactyla | Bovidae | *Bos* | | *Bos taurus* |
| 122 | 0 | 0 | 0 | Mammalia | Artiodactyla | Suidae | *Sus* | | *Sus scrofa* |
| 0 | 0 | 0 | 120 | Mammalia | Primates | Hominidae | *Homo* | | *Human DNA* |
| 0 | 0 | 116 | 0 | Mammalia | Artiodactyla | Bovidae | *Bos* | | *Bos taurus* |
| 113 | 0 | 0 | 0 | Mammalia | Rodentia | Muridae | *Mus* | | *Mus musculus* |
| 91 | 0 | 0 | 0 | Mammalia | Primates | - | *Homo* | | *Human DNA* |
| 0 | 79 | 0 | 0 | Mammalia | Primates | - | *Homo* | | *Human DNA* |
| 0 | 0 | 69 | 0 | Mammalia | Artiodactyla | Bovidae | *Ovis* | | *-* |
| 65 | 0 | 0 | 0 | Mammalia | Rodentia | Sciuridae | *Sciurus* | | *Sciurus vulgaris* |
| 0 | 0 | 62 | 0 | Mammalia | Carnivora | Felidae | *Felis* | | *-* |
| 0 | 56 | 0 | 0 | Mammalia | Carnivora | Canidae | *Canis* | | *Canis lupus* |
| 0 | 0 | 0 | 45 | Mammalia | Artiodactyla | Cervidae | *Hydropotes* | | *Hydropotes inermis* |
| 0 | 0 | 29 | 0 | Mammalia | Rodentia | Muridae | *Mus* | | *Mus musculus* |
| 5 | 0 | 0 | 0 | Mammalia | Unkwon Mammal | | | | *Human DNA* |
